# Metabolic profil in a group of obese Moroccan children enrolled in schools in the city of Rabat

**DOI:** 10.11604/pamj.2014.19.377.3630

**Published:** 2014-12-12

**Authors:** Nezha Mouane, Imane Cherkaoui Dekkaki, Said Ettair, Toufik Meskini, Nabil Khalloufi, Aziz Bouklouze, Amina Barkat

**Affiliations:** 1 Equipe de Recherche en Nutrition et Sciences de l'Alimentation, Faculté de Médicine et de Pharmacie de Rabat, Université Mohammed V de Rabat, Maroc; 2 Faculté de Médicine et de Pharmacie de Rabat, Mniversité Mohammed V de Rabat, Maroc; 3 Laboratoire de biostatistiques et études épidémiologiques, Faculté de Médicine et de Pharmacie de Rabat, Université Mohammed V de Rabat, Maroc

**Keywords:** Obesity, child, abdominal obesity, blood pressure, metabolic syndrome

## Abstract

**Introduction:**

To determine the metabolic profile in a group of obese children in Morocco.

**Methods:**

The BMI, the waist circumference, the blood pressure and metabolic parameters in 73 children (37 obese and 36 normal) were compared.

**Results:**

80% of obese children had abdominal obesity (p <0.0001). For systolic blood pressure among children who have a higher value than the 95th percentile, 85.7% were obese and 14.3% children are normal children. For diastolic blood pressure, 83.34% of obese children had higher diastolic blood pressure values in the 95th percentile and 16.6% of normal children have a higher value than the 95th percentile (p=0.013). No obese child had hyperglycemia. The prevalence of metabolic syndrome was 21.6%.

**Conclusion:**

Obesity is number one risk of cardiovascular disease for children. Early detection can help for an appropriate care.

## Introduction

Obesity in children is a threat to public health due to the gradual and steady increase in prevalence in many countries. There is a risk of developing a constellation of metabolic disorders, hemodynamic and inflammatory diseases in association with cardiovascular disease. Plus, a significant number of obese children already suffer from metabolic syndrome [1]. For adults, metabolic syndrome is justified by at least three of five criteria: obesity, high blood pressure, high fasting serum triglycerides, low levels of high density lipoprotein cholesterol and glucose intolerance. Abdominal fat mass is an important factor in assessing the risk of cardiovascular disease and metabolic syndrome. An abdominal circumference greater than half the height of the child standing should be a warning among other risk factors [2]. Although in most cases, obese children do not express any complaints about medical, a number of metabolic abnormalities are found more frequently in groups of obese children when compared with groups non-obese children [3]. - Increase in blood pressure; - Increased total cholesterol, LDL; -cholesterol, LDL / HDL ratio; - Increased triglycerides; - Glucose intolerance, which in the most extreme cases can progress to type 2 diabetes. The goal in our study is to investigate the frequency of metabolic syndrome in a group of obese Moroccan children.

## Methods

**Population:** Rabat, Morocco′s capital is located in the north of the country on the Atlantic coast, an area of 118 km^2^. The population is about 627,000 people. The city has 81 public primary schools (31,305 students) and 63 private primary schools (14,269 students); 68% of children are enrolled in a public school against 32% in a private school. A survey was done between April and June 2010, on a sample of 1570 children in 4^th^ grade, from 23 primary schools in Rabat selected randomly. The medium age was 9.7 ± 0.95 years. The prevalence of overweight including obesity was 8.7% [4]. After the approval of the Ethics Committee of the Faculty of Medicine and Pharmacy in Rabat, and the parental consent of the children drawen, 73 children belonging to these schools (37 obese and 36 normal) were reviewed for clinical consultation and biological sample.

**Method:** we used in our study, the National Cholesterol Education Program Adult Treatement Panel III definition (NCEP ATP III), according to whish, at least three of the following five criteria must be found in the diagnosis of metabolic syndrome: Abdominal obesity: Waist circumference greater than 90th percentile, according to the French references to hide Rolland et al [5]; Low HDL cholesterol: HDL-cholesterol below the 10th percentile or 1.03 mmol/ l using the average of the 10th percentile according to the National Cholesterol Education Program (NCEP) Report of the expert panel on Blood Cholesterol Levels in Children and Teenagers [6]; High triglycérides: plasmatic triglycérides above 90th percentile or >1,24 mmol/l (Average of 90° percentile according to the age) [6]; High blood pressure: systolic blood pressure >95th percentile or diastolic blood pressure > 95th percentile [7]; Impaired glucose tolerance: fasting glucose > 6.1 mmol/l (1.09g/l) [8].

**Clinical evaluation:** weight and height: children were weighed with scales, (150 ± 0.1 kg) (Seca 750, Germany). height was taken up with a stadiometer wooden strip of metal 2 meters ± 0.1cm, equipped with a headrest movable horizontal (Seca, Germany) without shoes and underwear [9]. The body mass index (BMI) was calculated by dividing weight (kg) by height (m) squared (in kg / m^2^). For the classification of BMI, we used the reference curves of the WHO 2007 subjects aged from 5 to 19 years old defined as follows [10]: Overweight: > + 1 Standard Deviation (equivalent to BMI 25 kg/m^2^ à 19 years); Obesity: > + 2 Standard Deviation (equivalent to BMI 30 kg/m^2^ à 19 years); Thinness: < - 2 Standard Deviation; - Severe Thinness: < - 3 Standard Deviation.

**The waist circumference:** it was measured after normal expiration using a flexible tape and non-elastic of 2 meters. Blood pressure: Blood pressure was measured using a sphygmomanometer Mercury with a cuff adapted to children, after 5 min rest in the supine position. Considered hypertensive children are those whose blood pressure exceeds the 95th percentile level. (Depending on height, sex and age) [7, 11–13]. The same material was used for all children by a pediatrician trained in advance awareness and action on the progress of the study.

**Biologicale evaluation:** samples were done on children fasting for at least 12 hours. Obese children only received the assay of HDL cholesterol and triglyceride levels, while the glucose level was performed for all children (obese and normal). Serum levels of glucose were determined according to hexokinase enzymatic method. Serum levels of: cholesterol, triglycerides and HDL were determined according to colorimetric enzymatic method

**Statistical analysis:** a group of obese children was analyzed. Then we proceed to the comparison of a group of obese children and group normal children. Statistics were performed on the software Statistical Package for Social Sciences (SPSS) version 18.0. Quantitative data are expressed as mean ± standard deviation and qualitative ones as a percentage. Correlations between two parameters were estimated by the Pearson correlation coefficient. The average parameters were compared using parametric tests for comparison of averages (Student′s t test). Comparison of two percentages was made by the Chi2 test or Fisher exact test (in case the conditions for the Chi2 test were not met). For all tests, the risk of error granted was set at p= 0.05.

## Results

### Anthropometry, blood pressure and biology

Our study included 73 children, including 36 girls and 37 boys. Our population consists of 36 normal children and 37 obese children. The average age was 11.02 ± 0.73, going from 8 to 13.6 years old. The average weight was 45.07 ± 13.80 kg. Table 1 shows the main anthropometric characteristics, blood pressure and the results of the amount of normal and obese children. All anthropometric parameters and blood pressure were significantly higher in obese children compared to normal children (p<0.05). There is no significant relationship between the average of the level glucose and BMI (p = 0.631).

**Table 1:** Anthropometric characteristics, biological and blood pressure of normal and obese children

	Normal (n=36)	Obese (n= 37)	P
Age ( years)	11.01 ± 0.54	11.04 ± 0.88	NS
Weight (kg)	32.89 ± 4.30	56.93 ± 8.37	< 0.0001
Height (m)	1.37 ± 0.74	1.48 ± 0.69	< 0.0001
BMI (kg/m^2^)	17.44 ± 0.93	25.66 ± 2.72	< 0.0001
Waist Circumference (cm)	68.14 ± 6.45	79.05 ± 6.33	< .0001
Systolic blood pressure (mm Hg)	11.05 ± 1.09	11.89 ± 1.19	0.002
Diastolic blood pressure (mm Hg)	6.15 ± 1.04	7.32 ± 0.78	< .0001
Fasting glucose (mmo/l)	4.66 ± 0.75	4.60 ± 0.36	0.704
Fasting glucose (g/l)	0.84 ± 0.13	0.82 ± 0.65	0.626

BMI Body Mass Index; NS: not significant; Result expressed as Mean ± Standard deviation

### Frequency of metabolic syndrome in obese children

**Figure 1:**
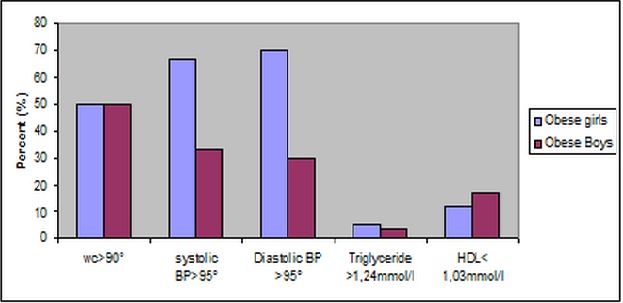
The three criteria of metabolic syndrome observed in obese boys and girls

Metabolic syndrome criteria have been investigated in 37 obese children (49% girls and 51% obese boys) with an average age of 11.04 ± 0.88 years old. The average BMI was respectively 26.81 ± 3 kg / m^2^ and 24.50 ± 1.82 kg / m^2^. Data by sex are detailed in Table 2. Figure 1 shows the frequency of metabolic syndrome criteria in obese children. Abdominal obesity is observed in all obese girls (49% of obese children). For boys, only one child has a waist circumference less than 90th percentile (not significant). 16% of obese children with systolic hypertension prevalence was two times higher among girls than boys (67% vs 33%). The average systolic blood pressure was (12.32 ± 1.34 vs 11.48 ± 0.89 p=0.031). For diastolic blood pressure 27% of obese children are hypertensive (70% girls against 30% of boys). Glucose intolerance was observed in any obese child. 8% of obese children had high triglycerides and low HDL cholesterol.

**Table 2:** Clinical and biological characteristics in obese children by gender

	Obese girls (n =18)	Obese boys (n =19)	P
Age ( years)	11.24 ± 0.88	10.86 ± 0.88	0.198
Weight (kg)	61.64 ± 8.73	52.47 ± 0.88	<0.0001
Height(m)	1.51 ± 0.05	1.46 ± 0.07	0.035
BMI (kg/m^2^)	26.81 ± 3	24.50 ± 1.82	0.005
Waist Circumference (cm)	80.08 ± 7.97	78.00 ± 4.25	0.356
Systolic blood pressure (mmHg)	12.32 ± 1.34	11.48 ± 0.89	0.031
Diastilic blood pressure (mmHg)	7.51 ± 0.76	7.14 ± 0.77	0.159
Fasting glucise (mmo/l)	4.56 ± 0.39	4.63 ± 0.33	0.54
Triglycérides (mmol/l)	1.05 ± 0.28	1.11 ± 0.66	0.71
HDL cholesterol (mmo/l)	0.49 ± 0.10	0.46 ± 0.10	0.37

**Figure 2:**
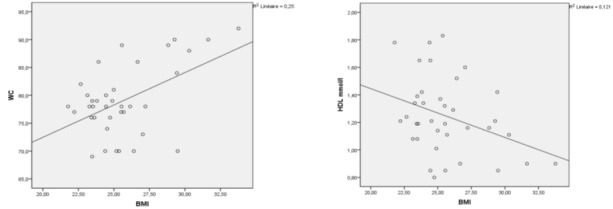
Correlation between the waist circumference and BMI and between HDL and BMI

**Figure 3:**
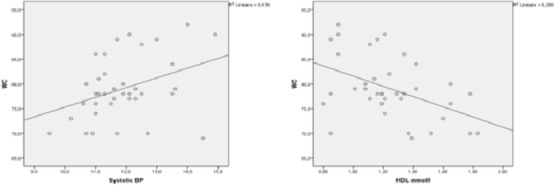
Correlation between waist circumference and systolic blood pressure and between waist circumference and HDL

**Figure 4:**
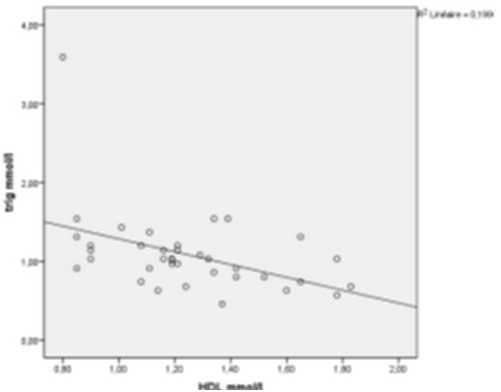
Correlation between HDL and Triglycerides

Figure 2, Figure 3 and Figure 4 show the correlation between (BMI / waist circumference), (BMI / HDL) (waist circumference / systolic blood pressure), (waist circumference / low HDL) and (triglycerides / low HDL). There′s a correlation between BMI and waist circumference (r=0.50 at 0.01, p=0.02), BMI is inversely related to HDL (r=- 0.34 at 0.05, p=0.03). The correlation between waist circumference and systolic blood pressure was significant (r=0.37 at 0.01, p=0.02) for waist circumference and HDL, there is a significant negative correlation (r=- 0.45 at 0.01, p=0.05). This is true for the correlation between triglycerides and HDL hypoglycemia, it is negative and highly significant (r=- 0.44 correlation threshold of 0.01, P=0.006).

### Comparison of obese and normal children

In our study we compared, waist circumference, blood pressure and blood glucose between normal and obese children (Table 3). 20% of normal children have abdominal obesity against 80% of obese children (p <0.0001). For systolic blood pressure among children who have a higher value than the 95th percentile, 85.7% were obese and 14.3% children were normal. For diastolic blood pressure, 84.34% of obese children had higher diastolic blood pressure value than 95th percentile and 16.6% of normal children have a higher value than the 95th percentile (p = 0.013). Hyperglycemia has been observed only in two normal children, no obese children showed hyperglycemia.

**Table 3:** Comparison of parameters of metabolic syndrome in obese and normal children

	Normal (%) n= 36	Obese (%) N=37	Total (%)	P
Waist circumference > 90	9 (20)	36 (80)	45 (100)	
Waist circumference < 90	27 (96.4)	1 (3.6)	28 (100)	<0.0001
Systolic blood pressure >95	1 (14.3)	6 (85.7)	7 (100)	
Systolic blood pressure < 95	35 (53.03)	31 (46.97)	66 (100)	**0.05**
Diastolic blood pressure > 95	2 (16.66)	10 (83.34)	12 (100)	
Diastolic blood pressure < 95	34 (55.73)	27 (44.27)	61(100)	0.013
Fasting glucose >6.1 mmol/l	2 (100)	0 (0)	2(100)	
Fasting glucose < 6.1 mmol/l	34(47.88)	37 (52.12)	71 (100)	0.146

## Discussion

Our study was performed on 73 children (36 normal children and 37 obese children). The children are enrolled in public primary schools in Rabat. The objective of the study is to investigate the frequency of metabolic syndrome in a group of obese children in Morocco.

The major risk of obese children is not only to remain obese when they become adult, but also and especially to be the cause or aggravate many diseases that are associated with obesity (cardiovascular disease, diabetes …) [14]. Children whose waist circumference exceeds 90th percentile are more likely to combine several risk factors for cardiovascular disease than those whose waist is below this threshold value [15]. According to our results, there′s a close relationship between BMI and waist circumference (p 16, 17]. There is a significant relationship between BMI and the level of systolic and diastolic blood pressure.(P=0.05 and p=0.013).

A significant correlation was found in our study (between waist circumference and systolic blood pressure (r=0.37 at 0.01, p=0.02). The association between obesity and high blood pressure in children has been shown in numerous studies in industrialized countries [18, 19]. With the increasing number of obese children, we can expect an increase in the incidence of high blood pressure in this class age. It is important that families, society and school support good prevention (diet and regular physical activity). As children of parents with high blood pressure or obese tend to have higher blood pressure values (either through genetic predisposition or an “unhealthy lifestyle” in the family), these children should benefit from specific preventive measures.

Our study showed an increased risk of hypertension (especially systolic) in obese girls, our results are comparable to a study in Mexico [20] and opposite to a study in obese adolescents in Algeria where the prevalence of hypertension was two times higher in boys than girls [21]. Glucose intolerance is much less common, being in only 10-15% of obese children [22]. Type 2 diabetes, however, is quite exceptional. Indeed, only children with a strong genetic predisposition to diabetes (children of Asian origin, and to a lesser extent, those genetically from Black Africa) can reveal the disease if they become obese. Children of European or North African, obesity does not complicate diabetes before adulthood [23]. Only two normal children out of the 73 children in our population had elevated fasting blood glucose. The prevalence of impaired fasting glucose was very low and relatively similar to that of other European series [23, 24].

For the metabolic syndrome in children, we chose to use the NCEP-ATPIII definition because it is the most commonly accepted definition in pediatrics. It also allows us to compare our results to other European studies with a neighbor definition [25, 26]. Morocco, a country in transition economic, social and cultural experiencing urbanization increasingly causing increased lifestyle changes similar to neighboring European countries such as France, Spain or Italy.

The highest prevalence after the waist obese children in our population was high blood pressure. The lowest prevalence were those of high triglycerides and low HDL cholesterol (8%). Even if our sample is small (37 obese children), our results showed that these children are predisposed to metabolic syndrome, since 49% of children are one criteria, and 27% are two criteria. There was a prevalence of metabolic syndrome by 22%. Our results are similar to those of Turkey; the prevalence of metabolic syndrome in children is 27.2% [27]. However, they are much lower than those reported in adolescents or American Indian prevalence of metabolic syndrome was 36% [28]. These data are hard to compare given the heterogeneity of the definition used to determine the metabolic syndrome in children and socio-demographic conditions of each population.

## Conclusion

Obesity in childhood and adolescence presents an immediate risk of co-morbidities and risk of adult obesity and persistent occurrence of cardio-metabolic complications. Our study has shown that waist circumference is predictive of risk of high blood pressure. Research of metabolic syndrome in obese children should be part of their care. Reducing the waist circumference is a goal to aim at the implementation of the project management of these children. Prevention measures must be in place soon that age.
